# Application of Fenton’s Reaction for Removal of Organic Matter from Groundwater

**DOI:** 10.3390/molecules29215150

**Published:** 2024-10-31

**Authors:** Izabela Krupińska

**Affiliations:** Faculty of Engineering and Technical Sciences, Institute of Environmental Engineering, University of Zielona Góra, 15 Prof. Z. Szafrana St, 65-417 Zielona Góra, Poland; i.krupinska@iis.uz.zgora.pl; Tel.: +48-68-3282560

**Keywords:** Fenton’s process, natural organic matter, disinfection by-products, groundwater

## Abstract

In this study, the effectiveness of the Fenton process in removing natural organic matter (NOM) from groundwater was investigated. The subject of this study is groundwater characterised by increased content of NOM and iron (II) compounds. In laboratory-scale studies, the influence of the ratio of concentrations of Fe(II) ions, which are naturally occurring in groundwater, to hydrogen peroxide (H_2_O_2_) as well as oxidation time and pH on the removal efficiency of organic matter was determined. Indicators such as total organic carbon (TOC), dissolved organic carbon (DOC), UV absorbance at 254 nm (UV_254_), UV absorbance at 272 nm (UV_272_), and specific UV absorbance (SUVA_254_) were used to quantitatively and qualitatively assess the organic substances present in the raw water and after oxidation with Fenton’s reagent. Analysis of the results obtained showed that the highest removal efficiency of organic substances in the deep oxidation process using the Fenton reaction was obtained for a concentration ratio of Fe(II) to H_2_O_2_ = 1:5. Acidification of the water samples to a pH of about 4 and extending the oxidation time to 30 min significantly increased the removal efficiency of organic substances including mainly dissolved organic substances containing aromatic rings. The organic substances containing aromatic rings, determined at a wavelength of 254 nm, were degraded to other organic intermediates.

## 1. Introduction

The sources of raw water for water treatment stations are surface water and groundwater, which very often contain above-normal concentrations of organic substances. Organic substances enter surface water with treated or untreated municipal or industrial wastewater and with surface runoff from urban, agricultural, or forestry areas. Organic substances may also be formed directly in the aquatic environment as a result of the biological activity of microorganisms. Groundwater also contains some organic matter. The main sources of organic matter are humus-rich soils from which humic substances are leached, and can also be landfill leachate, fertilisers, and plant protection products [1,2,3,4]. In the case of groundwater, the content of total organic carbon (TOC) varies, with values mostly ranging from 0.3 to 20 mgC/dm^3^. Dissolved organic carbon (DOC) in groundwater ranges from 0.2 to 15 mgC/dm^3^ with a median concentration of 0.7 mgC/dm^3^. The majority of all groundwaters have concentrations of DOC below 2 mgC/dm^3^. However, there are exceptions. These waters are coloured by humic substances and often have concentrations of DOC of 6 to 15 mgC/dm^3^ [5]. Organic substances, irrespective of their origin including natural organic matter (NOM), must be removed from the water during the treatment process to the extent that the quality standards for water intended for human consumption are met. According to the World Health Organization (WHO) guidelines for drinking water quality, the total organic carbon value should not exceed 5 mgC/L [6]. Organic substances must be removed prior to the disinfection process, as they are the main source of undesirable organohalogenated oxidation and disinfection products [7]. It has been confirmed that the oxidation with chlorine compounds of organic substances present in natural waters produces more than 700 compounds that are by-products of oxidation and disinfection, among which trihalomethanes (THMs) and haloacetic acids (HAAs) are the two most frequently formed and studied groups [8,9,10,11,12,13,14]. NOM is a complex and variable mixture of organic compounds, which therefore exhibits different reactivity in the formation of oxidation and disinfection by-products, depending on its origin and structure. NOM found in natural waters is composed of both hydrophobic and hydrophilic components, with hydrophobic acids usually representing the largest fraction, accounting for about 50% of the TOC in water [15,16,17,18]. These hydrophobic acids can be described as humic substances consisting of humic acids (Has), fulvic acids (Fas), and humin. Hydrophobic NOM is rich in aromatic carbon, phenolic structures, and conjugated double bonds, while hydrophilic NOM contains more aliphatic carbon and nitrogenous compounds such as carbohydrates, sugars, and amino acids. The hydrophobic NOM fraction is considered the most important precursor for the formation of THMs and HAAs [9,11,12,13]. However, some researchers report that the hydrophobic fraction is not always the main source of THM precursors. According to these authors, it is the hydrophilic/polar fraction that contains the highest amount of THM precursors of all NOM fractions [9,10]. The negative effects that can be caused by the presence of NOM, including the undesirable taste and colour of the water, means that its content must be minimised during water treatment. Coagulation, activated carbon adsorption, ion exchange, and membrane processes are used to remove organic matter from water, including NOM. Chemical oxidation with ozone used together with filtration through biologically active beds also leads to the removal of organic substances [1,2,3,4,19]. A significant increase in the removal efficiency of organic compounds, including humic substances, is also provided by advanced oxidation processes. In advanced oxidation processes (AOPs), highly reactive hydroxyl radicals (^•^OH) are generated, and one way of doing this type of oxidation is by using Fenton’s reagent. Fenton’s reagent is a mixture of hydrogen peroxide (H_2_O_2_) and iron (II) ions, catalysing the breakdown of H_2_O_2_ to hydroxyl radicals (^•^OH) according to reaction (1).

H_2_O_2_ + Fe^2+^ → Fe^3+^ + OH^−^ + ^•^OH (1)

The hydroxyl radicals formed have a high oxidation potential of 2.70 mV and act non-selectively on most organic compounds. The hypothesis that the Fenton reaction produces hydroxyl radicals has been proven by a number of techniques, including electron spin resonance spectroscopy (ESR) [20]. The amount of hydroxyl radicals is mainly determined by the pH of the water being treated, the doses of the chemical reactants, the oxidation time, and the temperature. The optimum pH value for most organic substances is between 2.8 and 3.0, and the oxidation time and doses of hydrogen peroxide and iron (II) ions depend on the type of compound being oxidised [21]. The pH range during the Fenton reaction is a major disadvantage of this process, as it requires acidification and subsequent neutralisation of the treated water. Hydrogen peroxide and Fe(II) ions dosed in excessive amounts can reduce the efficiency of the oxidation process by acting as hydroxyl radical scavengers, as shown in reaction (2).

Fe^2+^ + ^•^OH → Fe^3+^ + OH^−^ (2)

It has been shown that the generated hydroxyl radicals can oxidise organic substances through proton withdrawal, thus producing highly reactive organic radicals that can be further oxidised or degraded [22,23]. The chain reaction of the Fenton process proceeds in aqueous solutions through complex chemical reaction mechanisms involving, in addition to hydroxyl radicals, the formation of other radicals of varying reactivity. Hydroxyperoxy radicals are weaker oxidants compared to hydroxyl radicals, while superoxide radicals are weak reducing agents and nucleophiles [20,21,22,23]. Hydrogen peroxide, the main reagent for Fenton reactions, is a green reagent because the by-products are only water and oxygen. Moreover, hydrogen peroxide acts as a biocide/steriliser for removing microorganisms [24,25]. Fenton’s reagent has a number of advantages, such as the lack of formation of carcinogenic chlorinated organic compounds and the low price of the reagent components. It is also important that in full oxidation with Fenton’s reagent, organic compounds decompose to carbon dioxide and water or, in incomplete oxidation, to simpler molecules of lower molecular weight that are susceptible to biodegradation. The desired effect of advanced oxidation of organic pollutants using the Fenton reaction is to reduce the formation potential of THMs, as well as the unit amount of THMs (µg) produced from 1 mg of DOC [26]. The small-molecule oxidation products formed are less reactive in THM formation than the multi-molecule substances. The decrease in THM formation potential is accompanied by a decrease in the ultraviolet (UV) absorbance values measured at wavelength 254 nm as a result of the breaking of carbon–carbon double bonds. An increase in the proportion of the biodegradable fraction in the DOC was also found to range from 2.4 to 15.2% [27]. According to literature reports, the Fenton process has great potential for the treatment of NOM-rich water [18,28,29,30,31,32,33,34,35,36,37,38,39,40,41,42,43,44] and, according to some authors [8,21], the performance of this process is even better than coagulation with iron salts. However, despite the many advantages of using the Fenton reaction, it should be borne in mind that the use of deep oxidation methods may also result in the formation of undesirable oxidation products in the purified water. By-products formed as a result of AOPs may result in compounds more toxic than their parent molecules [45,46,47,48,49,50,51,52,53,54,55,56]. Among the oxidation by-products, a distinction is made between non-halogen organic compounds, halogen organic compounds, and inorganic by-products. The precursors of halogen and non-halogen organic compounds are organic substances and the inorganic by-products are mainly bromide ions. The type of oxidation by-products formed depends on the content, chemical structure, and properties of the precursors, the type and dose of oxidant, the contact time, and the pH of the water [47,48]. The Fenton reaction, like the ozonation process, can produce incomplete oxidation products of organic substrates present in the treated water, such as aldehydes, carboxylic acids and their esters, ketones, quinones, nitriles, and other oxidation intermediates, the type of which depends on the compound being oxidised [49,50]. If bromide ions are present in the treated water, organo-bromine compounds may also form. Due to their toxic, genotoxic, mutagenic, or carcinogenic effects, most organo-bromine compounds are considered hazardous to consumer health [45,55,57]. The results for groundwater with a TOC concentration of about 5 mgC/dm^3^ showed that during the oxidation process using the Fenton reaction and also as a result of ozonation, there was an increase in the concentration of total aldehydes, mainly formic and acetaldehyde, while the concentrations of glyoxal and methylglyoxal changed only slightly. Among the aldehydes identified in the groundwater after the oxidation process, formaldehyde and acetaldehyde accounted for 59%, glyoxal and methylglyoxal for 6%, and the other aldehydes accounted for the remainder [50]. It was also shown that the increase in concentration of individual aldehydes relative to their content in water before oxidation depended on the type of natural organic matter. In the case of formic and acetaldehyde, it was greater for humic acids and hydrophilic fractions, and in the case of glyoxal and methylglyoxal, it was greater for hydrophobic fractions. Carboxylic acids such as formic, acetic, oxalic and propionic acids can also be formed as by-products of the oxidation of organic substances during the Fenton process [48,49,50,51]. However, preceding the contact of organic compounds with free chlorine by their oxidation using the Fenton reaction or oxidation with ozone, clearly reduced the concentration of trihalomethanes (THMs), halogenoacetic acids (HAAs), and chloral hydrate formed during chlorination. However, it should be borne in mind that the oxidation by-products formed, formaldehyde and acetaldehyde, as well as glyoxal and methylglyoxal, also exhibit carcinogenic effects [55,56,57].

Iron (II) ions and hydrogen peroxide are required for the Fenton reaction. In groundwater with limited access to dissolved oxygen, iron occurs mainly as divalent iron Fe(II) [52]. In groundwater containing natural organic matter including humic substances, the rate of oxidation of iron (II) by dissolved oxygen is several times slower than in water containing no organic matter due to the stabilisation of iron (II) by organic substances. Organically complexed iron is not easily removed from groundwater, which is also confirmed by studies carried out by the author [53,54]. The author’s research [53,54] showed that in the case of groundwater from Quaternary formations due to the coexistence of natural organic matter and iron in groundwater, a certain part of the iron is present as iron–organic complexes in the form of colloids and/or dissolved complexes, and the water is characterised by increased colour intensity and turbidity. These waters create technological problems during their purification, due to the formation of iron–organic complexes. It is not possible to treat them with the conventional technological system of groundwater treatment: aeration, filtration, and disinfection; a process of coagulation with aluminium salts is required.

In this study, the efficiency of the removal of organic substances from groundwater of Quaternary formations characterised by increased natural organic matter content (TOC approx. 10 mgC/dm^3^) was determined using the Fenton reaction based on iron (II) ions present in the water. According to the WHO guidelines for drinking water quality, the total organic carbon value should not exceed 5 mgC/dm^3^ [6]. It was assumed that the deep oxidation process with Fenton’s reagent, with optimal technological parameters, should ensure high oxidation efficiency of organic substances, including substances containing aromatic rings, which, by stabilising iron, inhibit its removal and are also considered to be the main precursors of oxidation and disinfection by-products.

## 2. Results and Discussion

### 2.1. Ratios of Fe(II) to H_2_O_2_ Concentrations

**Figure 1:**
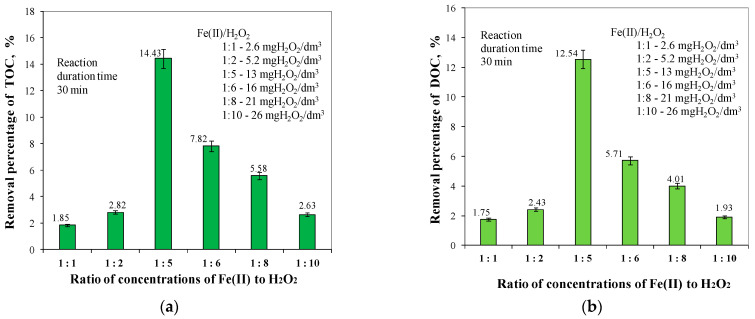
The effect of ratio concentrations of Fe(II) to H_2_O_2_ in the range of 1:1 to 1:10 on TOC (**a**) and DOC (**b**) removal efficiency.

In order to determine the efficiency of the removal of organic substances from groundwater by deep oxidation using the Fenton reaction, a technological study was carried out. In the first stage of this study, the effect of the ratio of Fe(II) ion concentrations to H_2_O_2_ on the efficiency of the removal of different fractions of organic substances at the natural pH of water of 7.30 was determined. The iron (II) concentration in the raw water was 2.6 mg/dm^3^. Figure 1 shows the effect of the ratio of Fe(II) ion concentrations to H_2_O_2_ changed from 1:1 to 1:10 on the efficiency of the removal of TOC and DOC from groundwater. Analysis of the results obtained showed that for a Fe(II) to H_2_O_2_ concentration ratio of 1:5, the highest removal efficiencies of both TOC and DOC were obtained, which were 14.43% (TOC) and 12.54% (DOC), respectively. For a Fe(II) to H_2_O_2_ concentration ratio of 1:5, the lowest concentrations of TOC and DOC were found in the treated water, which were 8.810 mgC/dm^3^ and 8.176 mgC/dm^3^, respectively. The concentration of TOC in water intended for human consumption should not exceed 5 mgC/dm^3^, while the permissible value of DOC is not specified [6,58].

Analysis of the results obtained also showed that in the Fe(II) to H_2_O_2_ concentration range of 1:6 to 1:10, the ^•^OH radical was found to be quenched by H_2_O_2_. Increasing the concentration ratio of Fe(II) to H_2_O_2_ from 1:5 to a value of 1:10 resulted in a decrease in the removal efficiency of both TOC and DOC by 11.80% and 10.61%, respectively. In general, increasing the dosage of H_2_O_2_ leads to an increase in the generation of hydroxyl radicals responsible for the degradation of organic matter, thereby increasing the removal efficiency of organic matter. This increase continues to a limited concentration of H_2_O_2_ as Fe^2+^, and then the removal efficiency decreases because of increased reactions between hydrogen peroxide and hydroxyl radicals and the scavenging effect of recombination of hydroxyl radicals at elevated hydrogen peroxide concentrations. In the Fenton system, H_2_O_2_ acts as both an ^•^OH radical generator and a quenching agent; excess H_2_O_2_ can act as a scavenger, trapping ^•^OH radicals resulting in reduced organic matter degradation efficiency [32,33]. According to other authors [8,21], the lower efficiency of the Fenton process in the case of raw groundwater may also be attributed to the nature of the NOM itself (e.g., structural differences), as well as to the influence of the matrix, especially the presence of bicarbonates and carbonates in the groundwater sample, which act as hydroxyl radical scavengers and reduce the efficiency of the process.

The lowest removal efficiencies of TOC (1.85%) and DOC (1.75%), within the error of the analysis, were obtained for a Fe(II) to H_2_O_2_ concentration ratio of 1:1. According to literature reports [21,33,45], the oxidation capacity of the Fenton process had a nonlinear correlation to the doses of [Fe^2+^]/[H_2_O_2_] due to the involvement of some unwanted side reactions at both high and low [Fe^2+^]/[H_2_O_2_] ratios. Figure 1 indicates the low mineralisation efficiency of TOC and DOC; one of the reasons for this low efficiency could be the pH of the reaction environment, which was 7.30. According to literature reports [45,46,55,56,57], pH is the primary factor determining the course and efficiency of the Fenton reaction. The strength of the oxidation potential of ^•^OH radicals changes with the pH value. In an acidic environment, the hydroxyl radical potential is 2.8 V. As the pH value increases, the radical potential decreases and reaches 1.5 V in an alkaline medium. In addition, the Fe(III) ions formed as a result of the Fenton reaction at pH above 5 transform into a colloidal form, which worsens the conditions for the decomposition of H_2_O_2_ into hydroxyl radicals. Because the ^•^OH generation rate and the pollutant reaction rate with ^•^OH are high-speed, the regeneration of Fe(II) is considered the rate-limiting step of both homogeneous and heterogeneous Fenton-like reactions.

During the ongoing technological research, the analytical scope for the determination of organic substances included, in addition to typical indicators such as TOC and DOC, the determination of UV absorbance measured at 254 and 272 nm, which determines fractions of organic compounds containing aromatic structures characterised by a high potential for the formation of oxidation and disinfection by-products [19].

**Figure 2:**
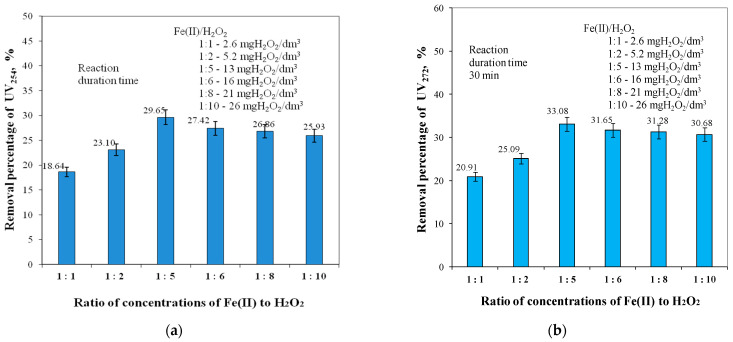
The effects of ratio concentrations of Fe(II) to H_2_O_2_ in the range of 1:1 to 1:10 on UV_254_ (**a**) and UV_272_ (**b**) removal efficiency.

Figure 2 shows the effect of the concentration ratio of Fe(II) ions to H_2_O_2_ varying from 1:1 to 1:10 on the removal efficiency of dissolved organic matter fractions containing aromatic rings (UV_254_ and UV_272_). The reduction efficiency of the absorbance measured at 254 nm varied from 18.64% to 29.65% and that of the absorbance measured at 272 nm from 20.91% to 33.08%. In general, the removal efficiency of the dissolved fractions of organic substances containing aromatic rings (UV_254_ and UV_272_) was much higher than that implied by the DOC reduction, indicating incomplete mineralisation of these organic pollutants (Figure 1 and Figure 2). According to literature reports [59,60,61,62,63,64,65], the Fenton reaction allows the oxidation of organic compounds by chain mechanisms. Subsequent oxidation steps initiated by this reaction produce radicals with weaker oxidizing potential, such as hydroperoxide (HO_2_^●^) or organic peroxy (ROO^●^), which react with specific functional groups of organic compounds. The reactivity of ^●^OH depends on the chemical structure of the compounds being oxidized. Aromatic compounds and unsaturated compounds have been shown to react faster with ^●^OH by electrophilic addition than saturated compounds, which react by hydrogen cleavage. The reaction rate constant increases as the number of hydrogen atoms in the molecule of a saturated compound increases. According to literature reports [13,14], the fractionation of organic compounds during oxidation increases their biodegradability and thus the possibility of their mineralisation to CO_2_ occurring with microorganisms.

**Figure 3:**
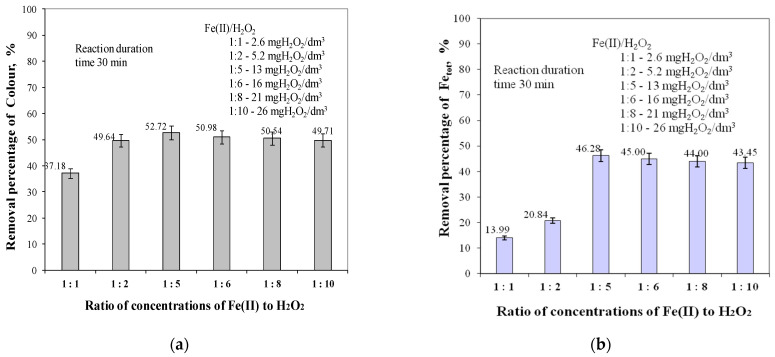
The effects of ratio concentrations of Fe(II) to H_2_O_2_ in the range of 1:1 to 1:10 on colour (**a**) and total iron (**b**) removal efficiency.

The analysis of the obtained test results presented in Figure 2 showed that, as in the case of TOC and DOC removal, the highest removal efficiencies of both UV_254_ and UV_272_ for aromatic ring-containing organics were obtained for a Fe(II) to H_2_O_2_ concentration ratio of 1:5, and these efficiencies were 29.65% for UV_254_ and 33.08% for UV_272_, respectively. Increasing the concentration ratio of Fe(II) to H_2_O_2_ to a value of 1:10 reduced the removal efficiencies of both UV_254_ and UV_272_ by 3.72% and 2.40%, respectively. The lowest UV_254_ (18.64%) and UV_272_ (20.91%) removal efficiencies were obtained for a Fe(II) to H_2_O_2_ concentration ratio of 1:1. The analysis of the test results in Figure 3 showed that the Fe(II) to H_2_O_2_ ion concentration ratio also affected the colour reduction and total iron removal efficiencies. As in the case of organic removal, the highest colour reduction (52.72%) and the highest total iron removal efficiency (46.28%) were obtained for a Fe(II) to H_2_O_2_ concentration ratio of 1:5.

The lowest colour (37.18%) and total iron (13.99%) reduction efficiencies were obtained for a Fe(II) to H_2_O_2_ concentration ratio of 1:1. According to literature reports, strongly oxidising hydroxyl radicals are produced during the Fenton process and Fe(II) ions are oxidised to Fe(III) ions, which are hydrolysed and precipitated as Fe(OH)_3_, as during coagulation with iron salts. The Fenton process can therefore also act as iron coagulation in water treatment [30,31,32,33,34].

The analysis of the obtained test results, presented in Figure 1, Figure 2 and Figure 3, showed that the highest efficiency in lowering all analysed water quality indicators, and especially in removing organic substances containing aromatic rings (Figure 2), was obtained for the ratio of Fe(II) to H_2_O_2_ concentration of 1:5.

### 2.2. Effect of pH in the Range of 2 to 8

According to literature reports, the mechanism of oxidation during the Fenton process is also influenced by the pH value, which determines the form of the oxidant and the oxidised compounds, and thus determines the different mechanisms of the oxidation reaction in acidic and basic environments [34]. Therefore, in a second series of technological studies, the effect of pH varied from 2 to 8 at a constant Fe(II) to H_2_O_2_ concentration ratio of 1:5 on the groundwater treatment efficiency.

**Figure 4:**
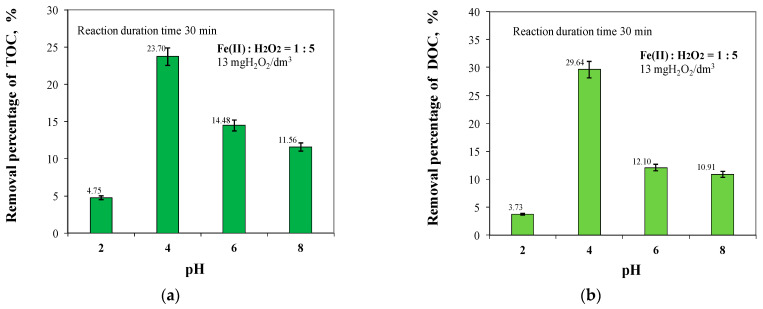
Effect of pH in the range of 2 to 8 on TOC (**a**) and DOC (**b**) removal efficiency.

Analysis of the test results in Figure 4 showed that the highest TOC and DOC removal efficiencies were obtained at pH = 4, with efficiencies of 23.70% and 29.64%, respectively. At pH = 4, the lowest concentrations of TOC and DOC were found in the treated water, which were 7.713 mgC/dm^3^ and 6.578 mgC/dm^3^, respectively. The concentration of TOC in water intended for human consumption should not exceed 5 mgC/dm^3^, while the permissible value of DOC is not specified [58]. Increasing the pH value from 4 to 8 reduced the removal efficiencies of both TOC and DOC by 12.14% and 18.73%, respectively. The lowest removal efficiencies for both total and dissolved organic carbon were obtained at pH = 2 at 4.75% and 3.73%, respectively. H_2_O_2_ reacts with protons existing in water in highly acidic conditions and forms oxonium ions. Since the H_2_O_2_ concentration required for hydroxyl radical generation decreases as a result of these reactions, the oxidation capacity of the Fenton process decreases in highly acidic conditions. At high pH values, the instability of hydrogen peroxide and ferrous ions causes decreases in the efficiency of the Fenton process. Indeed, Fe^2+^ remains dissolved even at neutral pH but Fe^3+^ disappears at pH ≥ 4 while forming ferric hydroxide sludge. Precipitation of Fe^2+^ as iron hydroxide can significantly reduce the catalytic decomposition of H_2_O_2_. Therefore, one of the most crucial operating parameters is pH, which controls the chemistry, the capacity to generate radicals, the catalyst behaviour, and the overall efficiency of the Fenton process [59,60,61,62,63,64,65].

**Figure 5:**
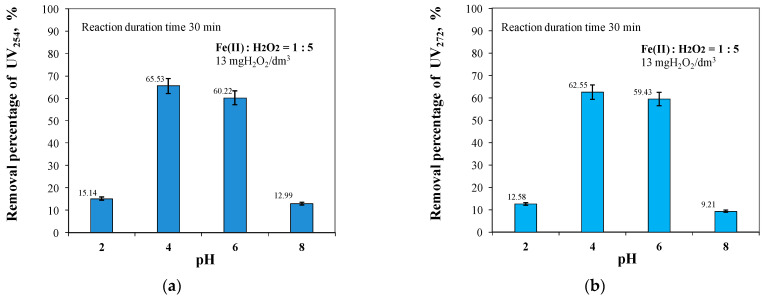
The effects of pH in the range of 2 to 8 on UV_254_ (**a**) and UV_272_ (**b**) removal efficiency.

As in the case of TOC and DOC removal, the pH of the raw water affected the removal efficiency of aromatic ring organics (UV_254_ and UV_272_). High removal efficiencies for aromatic ring-containing organics were obtained at both pH values of 4 and 6. The removal efficiencies for UV absorbance 254 at pH values of 4 and 6 were 65.53% and 60.22%, respectively, and for UV absorbance 272 were 62.55% and 59.43%, respectively (Figure 5).

Lowering the pH of the treated water to a value of 2 as well as increasing it to a value of 8 resulted in a large reduction in the removal efficiency of aromatic ring-containing organics (UV_254_ and UV_272_), as shown in Figure 5_._ In the case of UV_254_, the removal efficiency at pH = 2 compared to pH = 4 was low by about 50%, and at pH = 8 compared to pH = 6 by about 47%. In contrast, for UV_272_, the removal efficiencies at both pH = 2 and pH = 8 were low by about 50%. These results are in agreement with literature data, which indicate that the maximum catalytic activity in the Fenton process occurs in the pH range of 3 to 6 [28,33]. At pH values close to neutral, the efficiency of the process is much lower, as H_2_O_2_ has low stability at higher pH and then self-degrades to oxygen and water [32]. According to other authors [21,49], at pH above 5, the yield is lower due to the precipitation of iron (III) hydroxide, whereby the catalytic reaction of Fe(II) with oxidants is inhibited. Degradation of impurities in the Fenton reaction is therefore most effective in an acidic solution, which maintains the solubility of Fe(III) [18,21,32]. On the other hand, at pH values that are too low, the Fenton process may be less efficient due to the lower degradation rate of hydrogen peroxide [33]. According to literature reports [66], calcium peroxide (CaO_2_) could also be used to perform the Fenton reaction instead of liquid hydrogen, which is unstable. The use of CaO_2_ could be advantageous because, at a neutral pH (close to the pH of the groundwater), it allows a high percentage of H_2_O_2_ in the decomposition products. On the other hand, a way to increase the reactivity of iron ions could be to use the photo-Fenton process, which uses the classical Fenton reagent supported by ultraviolet radiation. During this process, the resulting Fe(III) ions are photo-reduced to Fe(II) ions, so that depletion of Fe(II) ions does not inhibit the process.

Analysis of the results obtained also showed that DOC fractions characterised by high aromatic ring content (UV_254_ and UV_272_) and thus high potential for the formation of oxidation and disinfection by-products were eliminated more efficiently than other organics. Various studies on advanced oxidation processes have shown that NOM decreases more when measured as UV_254_ than as dissolved organic carbon (DOC) [16,17,18,35,36,37,38,41], which is also confirmed by the results obtained. The greater reduction in UV_254_ values suggests that the chromophores in NOM macromolecules, which mostly consist of high molar mass aromatic rings, are rapidly decomposed into lower molar mass adducts that do not show absorbance at 254 nm. These adducts are less susceptible to attack by hydroxyl radicals and are therefore not completely mineralised [35,36,37,38,44].

**Figure 6:**
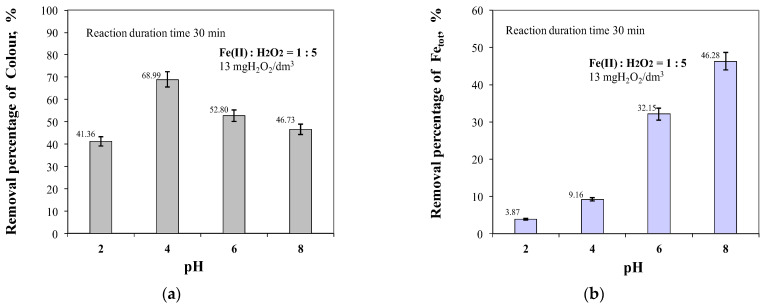
The effect of pH in the range of 2 to 8 on colour (**a**) and total iron (**b**) removal efficiency.

Figure 6 shows the influence of the pH value of the raw water on the effectiveness of colour reduction and the removal of total iron from the groundwater. Analysis of the test results obtained showed that the highest colour reduction efficiency, measured at a wavelength of 340 nm at which mainly organic substances containing chromophore groups absorb [19], was obtained at pH = 4, which may suggest that mainly coloured organic compounds were removed at this pH value. Both lowering the pH of the treated water to a value of 2 and increasing it to a value of 8 resulted in a decrease in the removal efficiency of the coloured compounds (Figure 6). Analysis of the results also showed that the efficiency of the removal of total iron from water by oxidation and sedimentation processes increased with increasing pH value. The lowest iron removal efficiency was obtained at pH = 2 and was 3.87%, while at the highest value of pH = 8, the efficiency of total iron removal increased to 46.28%. Due to the precipitation of iron (III) hydroxide, the catalytic reaction of Fe(II) with the oxidant is inhibited.

In raw water and after the pH correction process and oxidation with Fenton’s reagent, the value of the SUVA_254_ index was calculated as the ratio of UV absorbance measured at 254 nm to DOC concentration. The value of the SUVA_254_ index was determined because it is considered an indicator of the reactivity of dissolved organic substances that correlates well with the formation of oxidation and disinfection by-products and qualitatively determines the dissolved organic substances contained in the water [3,4,19].

**Figure 7:**
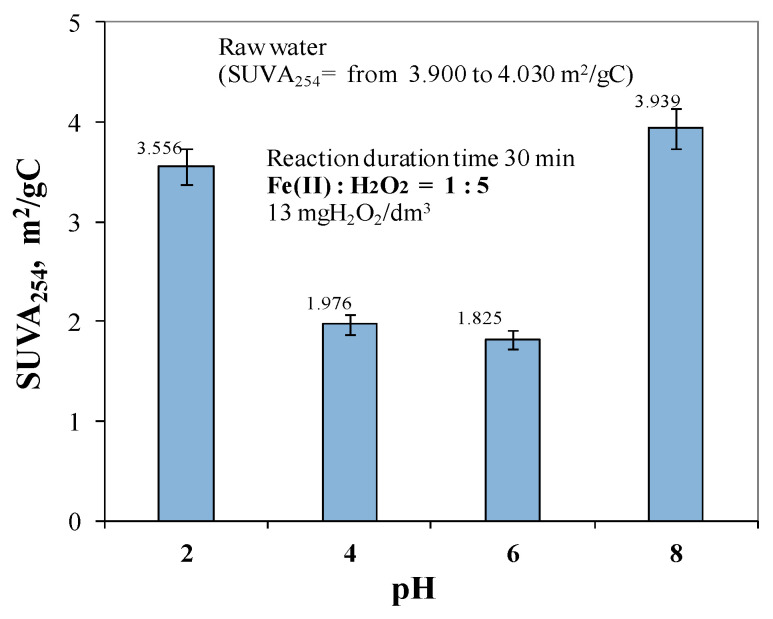
The effect of pH in the range of 2 to 8 on SUVA_254_ in treated water.

The calculated SUVA_254_ for the raw water ranged from 3.900 to 4.030 m^2^/gC, indicating that the water had a high content of hydrophobic, aromatic, and macromolecular DOC fractions, which are considered to be the main precursors of both THM and HAA. Figure 7 shows the effect of the pH value of the raw water during the oxidation process with Fenton’s reagent on the change in the SUVA_254_ index value in the treated water. The analysis of the obtained test results showed that when the pH was corrected to values of 2 and 8, the calculated value of SUVA_254_ was 3.556 and 3.939 m^2^/gC, indicating that both hydrophilic and hydrophobic as well as low and high molecular weight organic compounds were present in the treated water at these pH values; moreover, the criterion for the production of safe water for the consumer (SUVA_254_ ≤ 2 m^2^/gC) was not met due to the presence of organic precursors of oxidation and disinfection by-products [67].

In order to meet the criterion for the production of consumer-safe water, a pH adjustment to values 4 and 6 was required because, at these pH values, the calculated SUVA_254_ was 1.976 m^2^/gC and 1.825 m^2^/gC (SUVA_254_ ≤ 2 m^2^/gC), respectively.

The results obtained showed that the application of the Fenton process to the treatment of groundwater with elevated organic content can provide the removal of high-molecular-weight hydrophobic structures, which are considered to be the main precursors of both THM and HAA [18,37,38,42], and the parameter co-determining the efficiency of the Fenton reaction is the pH value at which the oxidation process took place. It was also found that during the removal of compounds affecting the colour intensity of the water and its level of organic contamination, the role of pH was far greater than the effect of the ratio of Fe(II) to H_2_O_2_ concentrations (Figure 1, Figure 2, Figure 3, Figure 4, Figure 5, Figure 6 and Figure 7).

### 2.3. Effect of Oxidation Time from 15 to 60 min

As the extension of the oxidation time should, according to literature reports [50], increase the efficiency of the oxidation of organic substances during the Fenton process, the effect of an oxidation time varying from 15 to 60 min on their removal efficiency was determined in a subsequent series of tests. The process of increasing the oxidation time was carried out at pH = 4 and a concentration ratio of Fe(II):H_2_O_2_ = 1:5 because the highest efficiency of groundwater treatment, and especially of organic matter removal, was obtained at these technological parameters. It should be borne in mind, however, that under technical conditions it is not always possible to extend the contact time significantly, which may result in the oxidation process not proceeding to completion and intermediate oxidation products being formed.

**Figure 8:**
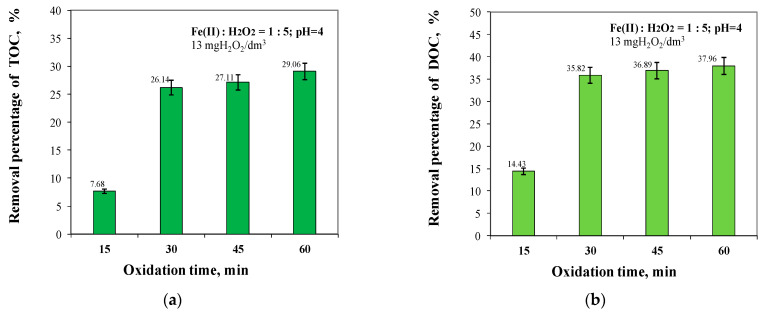
The effect of oxidation time from 15 to 60 min on TOC (**a**) and DOC (**b**) removal efficiency.

**Figure 9:**
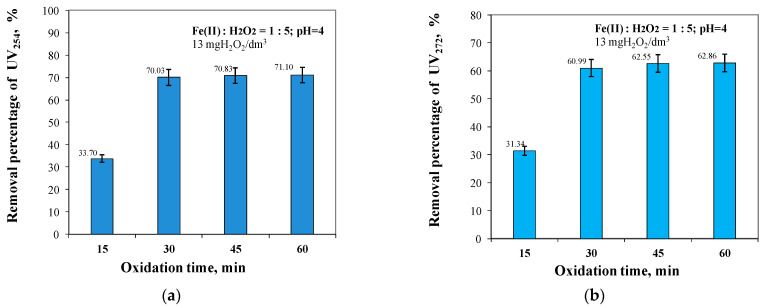
The effect of oxidation time from 15 to 60 min on UV_254_ (**a**) and UV_272_ (**b**) removal efficiency.

Analysis of the test results obtained, as shown in Figure 8 and Figure 9, showed that the greatest increase in organic removal efficiency resulted from an increase in oxidation time from 15 to 30 min for TOC by 18.46% and DOC by 21.39%, and for dissolved organic substances containing aromatic rings by 36.33% and 29.65% for UV_254_ and UV_272_, respectively.

For all organic fractions, the highest removal efficiencies were obtained for an oxidation time of 60 min, with a small increase in the removal efficiency compared to that obtained for an oxidation time of 30 min of 2.92% for TOC, 2.14% for DOC, 1.07% for UV_254_, and 1.87% for UV_272_, respectively (Figure 8 and Figure 9). For an oxidation time of 60 min, the concentration of TOC and DOC in the treated water was 7.172 mgC/dm^3^ and 5.800 mgC/dm^3^, respectively.

It was also shown that increasing the oxidation time from 15 to 30 min maximally increased the removal efficiency of dissolved organics containing aromatic rings, which are considered to be the main precursors of both THM and HAA (Figure 9). According to literature reports, the hydroxyl radical formed during the Fenton reaction first attacks the aromatic ring to form a hydroxycyclohexadienyl radical, which can be oxidised to phenol and then to catechol, hydroquinone, and benzoquinone, followed by ring cleavage [30,33,59,60,61,62].

**Figure 10:**
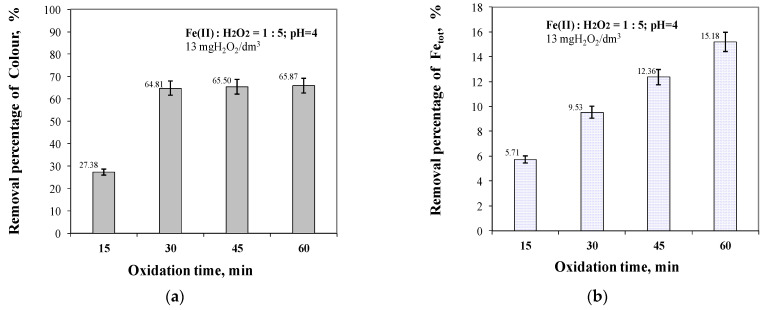
The effect of oxidation time from 15 to 60 min on colour (**a**) and total iron (**b**) removal efficiency.

The analysis of the test results shown in Figure 10 showed that, for coloured compounds, the highest removal efficiency, similar to organic compounds, was obtained for an oxidation time of 60 min, and that increasing the oxidation time from 15 to 30 min resulted in the highest increase in the removal efficiency for coloured compounds of about 37.43%.

**Figure 11:**
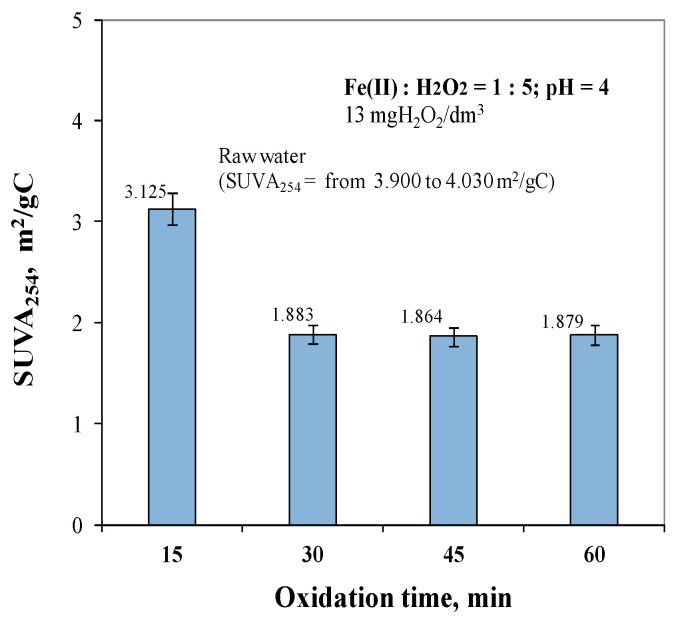
The effect of oxidation time from 15 to 60 min on SUVA_254_ in treated water.

It was also shown that in the case of the removal of total iron compounds (Figure 10), the highest removal efficiency was also obtained for an oxidation time of 60 min (15.18%). In the case of iron compounds, increasing the oxidation time from 15 to 30 min resulted in a 3.82% increase in removal efficiency, and from 30 to 60 min resulted in a 5.65% increase. Figure 11 shows the effect of oxidation time with Fenton’s reagent on the change in SUVA_254_ in the treated water.

Analysis of the obtained test results showed that comparable and lowest SUVA_254_ values were obtained for oxidation times of 30, 45, and 60 min and were 1.883 m^2^/gC, 1.864 m^2^/gC, and 1.879 m^2^/gC, respectively. For an oxidation time of 15 min, the highest SUVA_254_ value was obtained and was 3.125 m^2^/gC. In order to meet the criterion of producing water that is safe for the consumer (SUVA_254_ ≤ 2 m^2^/gC), an oxidation time of at least 30 min was required.

Although the SUVA_254_ index value indicates that the water is safe for consumers, the correlation analysis presented in Figure 8 and Figure 9 showed that the TOC removal efficiency (7.68–29.06%) was lower than the absorbance reduction efficiency measured at 254 nm during the Fenton reaction (33.70–71.10%). This phenomenon suggests the hypothesis that the organic substances containing aromatic rings, measured at 254 nm, were degraded to other organic intermediates.

**Figure 12:**
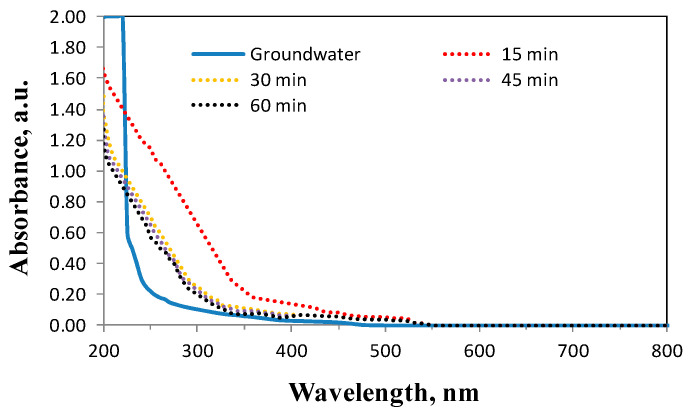
UV-Vis spectra in the Fenton reaction in conditions of Fe(II)/H_2_O_2_ = 1:5 (13 mgH_2_O_2_/dm^3^), pH = 4.

Therefore, further analysis was carried out to reveal the mechanism by analysing the UV-Vis spectra shown in Figure 12.

The UV-Vis spectra for the Fenton reaction at pH = 4, Fe(II)/H_2_O_2_ = 1:5, and reaction times from 15 to 60 min are shown in Figure 12. Analysis of the obtained results showed that the intensity of the spectral band characteristic of groundwater after the addition of H_2_O_2_ to the system decreased especially for the wavelength range from 200 up to about 250 nm, while a strongly absorbed spectral band in the range 200–550 nm was detected. It can be concluded that a large number of complex intermediates were generated with strong absorption in the UV-Vis range. According to literature reports [68,69,70], carboxylic acids absorb in the range of 190–230 nm. Absorbance in the rough neighbourhood of 260–320 nm is common for molecules containing a C=O group (such as ketones and aldehydes) and this corresponds to a (n→π*) transition [68].

It was also shown that the absorbance decreased sharply during the first 30 min of the reaction and then remained constant. According to Figure 9, the mineralisation yield of organic substances containing aromatic rings, strongly absorbing at 254 nm, reached about 70% after 30 min; however, absorption spectral bands in the ultraviolet regions still existed. It can therefore be concluded that the Fenton reaction is not a simple radical reaction. Combining with the theory of other researchers [68,69], the assumption was proposed that the large absorption spectrum band was generated by the reaction of Fe(II) and organic substances. Probably, mainly organic substances containing aromatic rings were partially oxidised by electron transfer in the complex compound. This confirms the reports that the efficiency of the Fenton process is largely determined by the nature of the organic compounds being oxidised, their chemical structure, and the degree of aromaticity. According to literature reports [71,72,73], organic compounds containing aromatic rings are more easily oxidised in the classical Fenton process, while aliphatic ones require the additional use of UV lamps. NOM with higher molecular weight compounds is partially oxidized and transformed into smaller ones, such as e.g., aldehydes and carboxylic acids.

**Figure 13:**
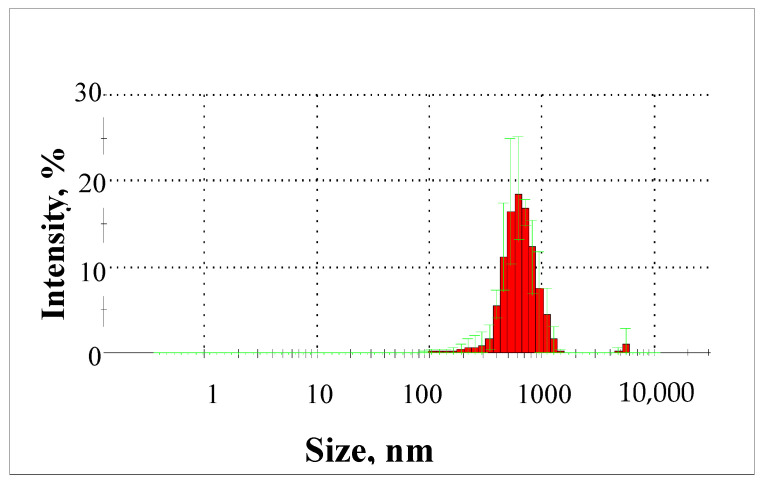
Particle size distribution from 190 to 6440 nm in groundwater.

Particle size measurements using a Zetasizer Nano analyser showed that particles in the raw groundwater ranged in size from 190 to 6440 nm (Figure 13).

Almost 86% of the particles present in the groundwater were particles with a diameter in the range of 531 to 955 nm.

**Figure 14:**
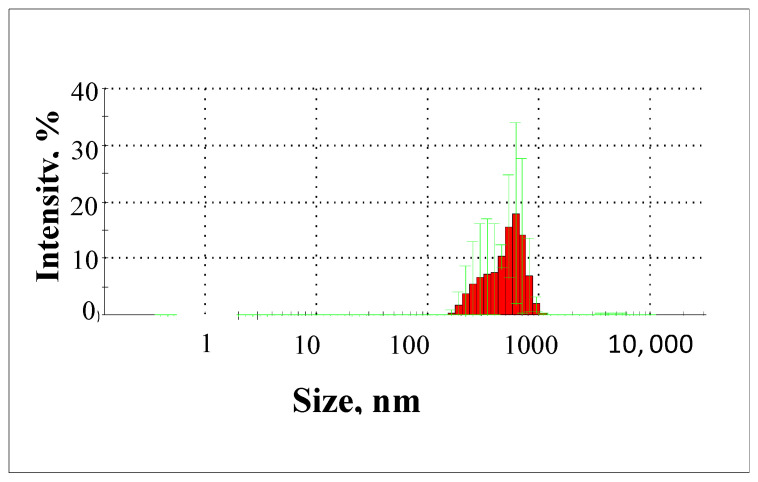
Particle size distribution from 140 to 955 nm in water after oxidation with Fenton’s reaction in conditions of Fe(II)/H_2_O_2_ = 1:5 (13 mgH_2_O_2_/dm^3^), pH = 4, and an oxidation time of 30 min.

In the water after the oxidation process using the Fenton reaction for Fe(II)/H_2_O_2_ = 1:5, pH = 4, and an oxidation time of 30 min, particles in the range of 140 to 955 nm were present (Figure 14). Almost 70% of the particles present in the groundwater after the oxidation process using the Fenton reaction were particles with diameters in the range of 140 to 390 nm. Analysis of the results obtained showed that the Fenton reaction resulted in the partial oxidation of particles with diameters above 531 nm, while the proportion of particles with diameters below 390 nm increased after the oxidation process in the treated water. This means that particles with a diameter above 531 nm were more reactive towards hydroxyl radicals during the Fenton reaction.

## 3. Materials and Methods

### 3.1. Water Samples

The subject of this study is groundwater from Quaternary formations extracted for water supply purposes characterised by elevated contents of iron (II) compounds and organic substances, including organic substances containing aromatic rings. Iron (II) compounds ranging from 2.542 to 2.677 mgFe/dm^3^, total iron from 3.431 to 3.537 mgFe/dm^3^, and iron (III) from 0.860 to 0.889 mgFe/dm^3^ were present in the groundwater. TOC reached values from 10.110 to 10.290 mgC/dm^3^, DOC from 9.150 to 9.349 mgC/dm^3^, UV_254_ absorption from 35.65 to 37.71 m^−1^, and UV_272_ from 28.43 to 32.04 m^−1^, which indicates that organic substances containing aromatic rings, which are characterised by a high potential of forming oxidation or disinfection by-products, were present among the dissolved substances in the purified water. The tested water was also characterised by increased colour from 20.31 to 27.54 mgPt/dm^3^ and turbidity from 3.73 to 5.53 NTU. The calculated SUVA_254_ varied from 3.90 to 4.03 m^2^/gC, indicating that the purified water contained a mixture of hydrophobic and hydrophilic humic substances and other natural organic compounds of both small and large molecular weights [19].

**Figure 15:**
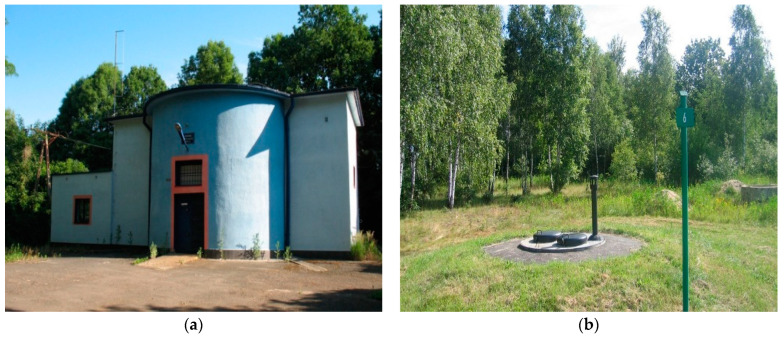
Groundwater intake (**a**), leveller well (**b**).

The levelling groundwater intake is located in the area of the Warsaw–Berlin Proglacial Valley, approximately 4 km from the river’s shoreline, in the zone of the Oder’s old river bed. It consists of 22 wells, 18 to 30 m deep, located near the peat bogs. Figure 15 shows the groundwater intake and one of the leveller wells.

Underground water samples for testing were taken directly from a test cock placed on the water discharge pipe. Simax 1 dm^3^ glass bottles with a screw cap were used for water sampling. The glass bottles were filled using a PVC laboratory hose until the water in the bottle was replaced twice. The bottles were filled completely with the sampled water, taking care that no air bubbles remained while closing the cap. The water samples were transported to the laboratory in a refrigerator at approximately 4 °C.

### 3.2. Technological Research

**Figure 16:**
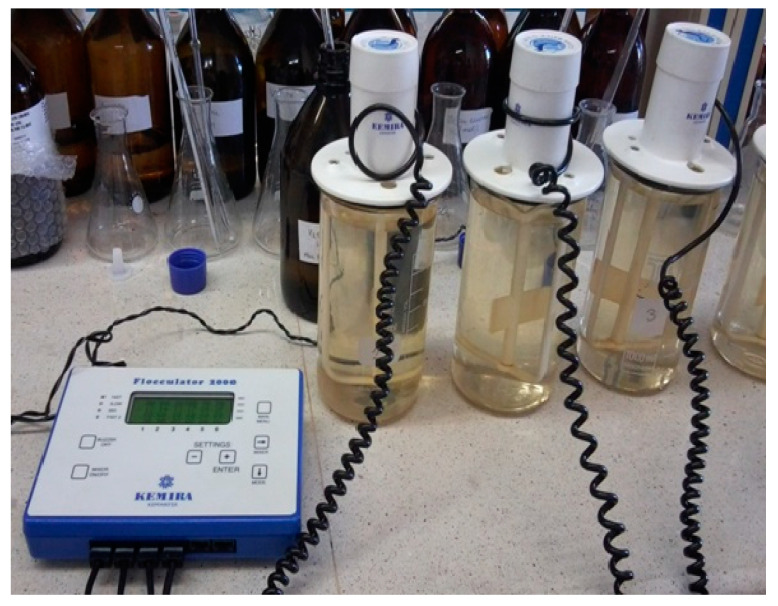
Oxidation process using Fenton’s reaction.

In laboratory-scale studies, the effect of the ratio of concentrations of Fe(II) ions naturally occurring in groundwater to hydrogen peroxide, as well as oxidation time and pH, on the efficiency of the removal of organic substances from groundwater was determined. The first series of tests determined the effect of the ratio of Fe(II) to hydrogen peroxide concentrations varied from 1:1 to 1:10, the second series of tests determined the effect of pH varied from 2 to 8, and the third series of tests determined the effect of oxidation time from 15 to 60 min on the efficiency of organic matter removal from groundwater. Prior to the technological tests, the concentration of iron (II) in the raw water was determined, as the dose of hydrogen peroxide was determined precisely because of its concentration. Oxidation was carried out with Fenton’s reagent with a mass ratio of Fe(II)/H_2_O_2_ of 1:1, 1:2, 1:5, 1:6, 1:8, and 1:10. Before the appropriate amount of hydrogen peroxide was introduced into the groundwater, a 30% solution of H_2_O_2_ was diluted 10 times to obtain a 3% solution. When adding hydrogen peroxide to the groundwater, a rapid stirring of 180 rpm was used (to completely mix the water and oxidant), followed by slow stirring at an intensity of 30 rpm. The oxidation time was varied from 15 to 60 min. During the second series of tests, 0.1 M hydrochloric acid or 0.1 M sodium hydroxide was dosed into the raw water to correct the pH between 2 and 8. It is well known that the optimum pH of the Fenton reaction is 2.8–3.0 for the removal of organic matter [32,33], but this study also investigated the performance of the Fenton process at pH values close to that of natural water. The oxidation process using the Fenton reaction was carried out using a Flocculator 2000 from Kemira Kemwater (Helsingborg, Sweden) Figure 16. Each process was carried out in triplicate.

#### Reagents and Materials

Hydrogen peroxide (H_2_O_2_ solution 30%, pure for analysis, density (20 °C) 1.112 g/cm^3^, and residue after evaporation max. 0.005%) was sourced from Chempur (Karlsruhe, Germany). Hydrochloric acid (HCl 0.1 M, analytical ampoule contains 3.646 g HCl) and sodium hydroxide (NaOH 0.1 M, analytical ampoule contains 4.000 g NaOH) were sourced from Chempur.

### 3.3. Analytical Methods

The physical–chemical composition of both the raw water as well as treated water was determined according to the International Standard methods. The colour (according to the Pt scale), total iron, and iron (II) concentrations were determined with the UV-Vis spectrophotometer Cary 3500 (Cary, Santa Clara, CA, USA). Iron (II) and total iron were measured using the 1,10 phenanthroline method [74]. Iron (III) concentrations were calculated as the difference between total iron and iron (II). The organic substances concentration was monitored by measuring the TOC, DOC, and UV absorbance at 254 and 272 nm [19,75,76,77]. The TOC and DOC were measured using the thermal method and a multi N/C 3100 DUO Analytik Jena TOC Analyzer (Jena, Thuringia, Germany). DOC was analysed by the multi N/C 3100 DUO Analytik Jena TOC Analyzer after filtration through 0.45 µm pore diameter membranes.

The removal percentage TOC was measured using Equation (3):

(3) $$\mathrm{Removal}\;(\%)\;\mathrm{TOC}=\frac{C_{o}-C_{f}}{C_{o}}\cdot 100$$

where Removal (%) is the TOC removal percentage (%), (*C_o_*) is the value of TOC before treatment (mgC/dm^3^), and final concentration (*C_f_*) is the value of the final TOC (mgC/dm^3^) [78]. Based on Equation (3), the removal efficiency of all analysed water quality indicators was also calculated.

UV absorbance at 254 nm (UV_254_) and at 272 nm (UV_272_) was measured by a UV-Vis spectrophotometer Cary 3500 (Santa Clara, CA, USA) using a quartz cell with a 1 cm path length after filtration through a 0.45 µm membrane. On the basis of our own research, it was shown that an increase in the amount of iron (III) ions in water causes a linear increase in absorbance measured at λ = 254 nm and at λ = 272 nm (A_254_ = 0.060[(Fe(III)] + 0.002, cm^−1^; A_272_ = 0.065[(Fe(III)] + 0.005, cm^−1^). Therefore, the results of the absorbance measured at 254 nm and at 272 were corrected taking into account the concentration of Fe(III) in the sample. There was no effect of H_2_O_2_ on the absorbance measured at 254 and 272 nm.

DOC and UV_254_ are used in the calculation of the specific UV absorbance (SUVA_254_) as follows:

SUVA_254_ = UV_254_ nm/DOC (m^2^/gC) (4)

where SUVA is the specific UV absorbance at 254 nm (m^−1^) and DOC is dissolved organic carbon (gC/m^3^) [3,19]. The pH and temperature of the raw water and the purified water were determined with a WTW 3620 IDS SETC (Troistedt, Germany) with a combination pH electrode with temperature corrections. UV/Vis spectra were recorded by using a UV-Vis spectrophotometer Cary 3500 (Santa Clara, CA, USA). The samples were scanned by spectrophotometer at a wavelength ranging from 200 to 1000 nm.

In the water, the particle size was also measured using the Zetasizer Nano Analyzer (Malvern Panalytical, Malvern, UK). The Zetasizer Nano Analyzer measures particle size using the dynamic light scattering (DLS—dynamic light scattering) process, also known as photon correlation spectroscopy (PCS—photon correlation spectroscopy), which measures Brown’s motion and calculates particle size on this basis. The intensity of the fluctuation of the scattered laser light that the particles are illuminated by is analysed.

## 4. Conclusions

Despite decades of experience and continuous advances in knowledge, the treatment of water for human consumption containing increased amounts of organic matter poses technological problems. In the case of groundwater from Quaternary formations containing increased amounts of iron and organic matter, it is not sufficient to use a conventional technological system for groundwater treatment such as aeration, sedimentation, and filtration, but a coagulation process is recommended. Increasing the removal efficiency of organic substances, including organic substances containing aromatic rings, hindering the removal of iron and being precursors of oxidation by-products of disinfection with chlorine compounds, can also be provided by advanced oxidation processes, and one way to do this can be the use of the Fenton reaction. This study summarises the key findings from laboratory studies on the application of the Fenton reaction to the removal of organic matter from groundwater from Quaternary formations, using iron (II) ions present in the groundwater. The effects of iron (II) ion/hydrogen peroxide concentration ratios ranging from 1:1 to 1:10, pH varying from 2 to 8, and oxidation time varying from 15 to 60 min on organic removal efficiency were investigated.

The analysis of the obtained results of the application of the Fenton reaction for the purification of groundwater from Quaternary formations, characterised by a high content of hydrophobic and aromatic and macromolecular DOC fractions, showed that the highest efficiency of its purification was obtained for a ratio of Fe(II) to H_2_O_2_ concentrations of 1:5. The parameter co-determining the effectiveness of the Fenton reaction was the pH value at which the oxidation process took place, and the highest removal efficiency of all organic matter fractions was obtained at pH = 4. It was also found that during the removal of compounds affecting the intensity of the water colour and the level of organic contamination, the role of pH was far greater than the influence of the ratio of Fe(II) to H_2_O_2_ concentrations. The deep oxidation process using the Fenton reaction provided a high removal efficiency of dissolved organic substances containing aromatic rings (UV_254_ and UV_272_). Analysis of the results also showed that the TOC removal efficiency was lower than the absorbance reduction efficiency measured at 254 nm during the Fenton reaction. This phenomenon proposes the hypothesis that the organic substances containing aromatic rings, determined at 254 nm, were degraded to other organic intermediates, such as, e.g., aldehydes and ketones, and carboxylic acids could be present, i.e., compounds much more dangerous to the human body than those removed from groundwater. It was also shown that increasing the oxidation time from 15 to 30 min resulted in the greatest increase in the removal efficiency of organic substances, especially dissolved organic substances containing aromatic rings. Although an SUVA_254_ value of ≤2 m^2^/gC was obtained for a pH adjustment of 4 or 6 in the treated water, due to incomplete mineralisation of organic substances containing aromatic rings, the water was nevertheless not safe for the consumer, as evidenced by the analysis of the UV-Vis spectra.

The results obtained show that the application of the Fenton process for the treatment of groundwater from Quaternary formations with increased amounts of organic matter, using iron (II) ions naturally occurring in water, should be combined with other unit water treatment processes, such as, e.g., the activated carbon bed filtration process, due to the formation of oxidation by-products dangerous to water consumers. As the highest organic removal efficiency was achieved at pH = 4, alkalinisation will also be required to restore the carbonate–calcium balance of the treated water. It should also be borne in mind that organic substances should be removed from the water first and foremost and that methods of destroying them by chemical oxidation should be considered as a last resort.

## Data Availability

The original contributions presented in the study are included in the article, further inquiries can be directed to the author corresponding.
